# Supplementary material for: Inhomogeneity-driven multiform Spontaneous Hall Effect in conventional and unconventional superconductors
Source: Sci Technol Adv Mater. 2025 Aug 21;26(1):2546282. doi: 10.1080/14686996.2025.2546282 (PMC12418796; doi:10.1080/14686996.2025.2546282)
Supplement: Supplemental Material [file TSTA_A_2546282_SM4926.docx]

**SUPPLEMENTAL MATERIALS**

A) EXPERIMENTAL DETAILS


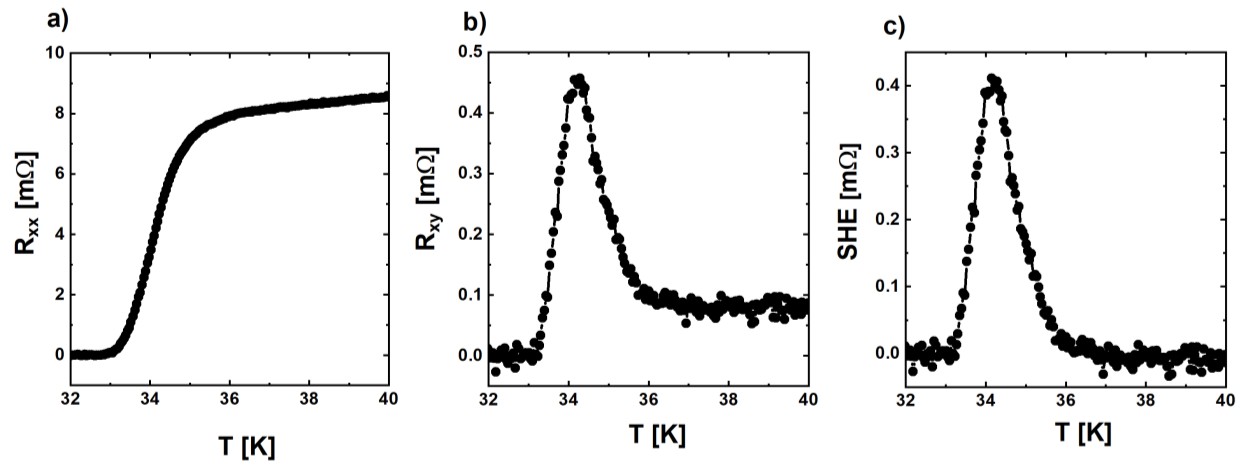


**Figure 7**: Typical experimentally measured signals of the SHE. (a): longitudinal resistance of a superconducting material. (b): transverse resistance measured in absence of a magnetic field. (c): SHE signal extracted as explained by equation 3.

Typical signals of the longitudinal Rxx resistance and the transverse Rxy resistance simultaneously measured are presented in fig. 7. The transverse resistance corresponds to the superimposition of the superconductive transition with a peak, developing across the transition itself, who is the focus of our debate. To extract the SH peak, the Rxx curve is subtracted to the transverse one, after a simple rescaling process, as given by the following equation:

𝑆𝐻𝐸 = 𝑅𝑥𝑦(𝑇) − 𝛼𝑅𝑥𝑥(𝑇) (1)

Where _𝛼 =_ 𝑅𝑥𝑦(𝑇>𝑇𝑐)

𝑅𝑥𝑥(𝑇>𝑇𝑐)


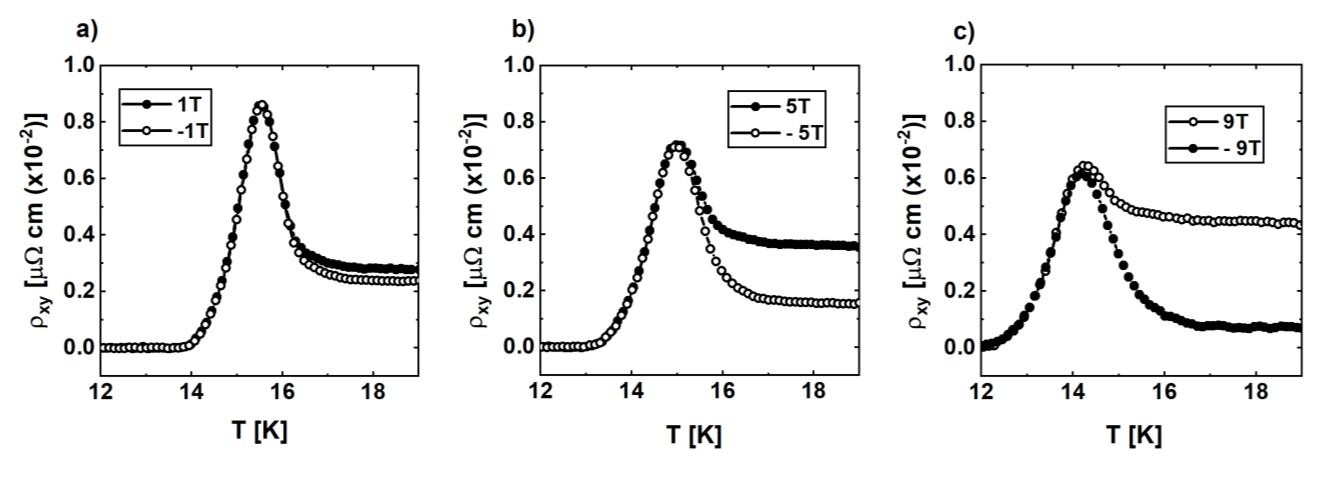


**Figure 8**: ρxy curves at positive (full dots) and negative (empty dots) magnetic fields (1T, 5T, 9T) of Fe(Se,Te) thin film - Hall bar 1.

Fig. 8 presents the transverse resistivity curves at applied magnetic fields across Hall bar 1 of the Fe(Se,Te) thin film. In particular, data at both positive (full dots) and negative (empty dots) magnetic fields are shown. The in-field behavior of ρxy demonstrates the superimposition of a symmetric signal (SHE) and an asymmetric one (standard Hall effect). Therefore, the SHE can be clearly extracted from ρxy, and distinguished from the standard Hall effect, in present.

B) FINITE ELEMENT SIMULATION DETAILS


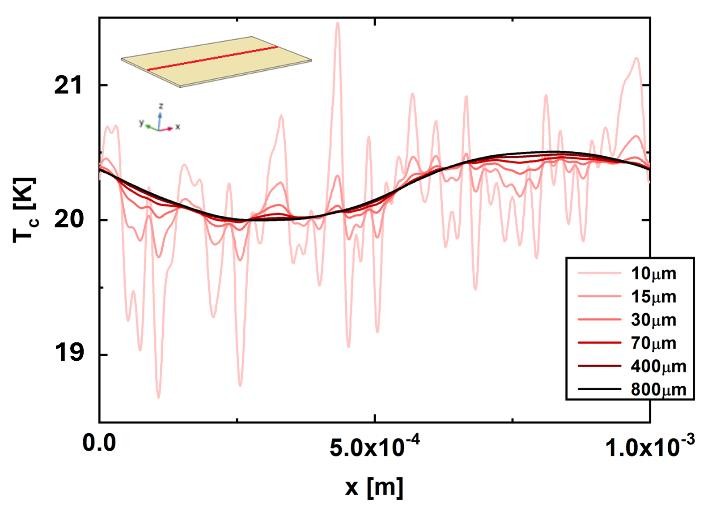


**Figure 9**: Profile of the Tc distribution around 20K along the x-direction of the sample for all the values of the disorder scale from 10 to 800 μm.

The spatial distribution of disorder in the sample is simulated using a critical temperature distribution centred around 20K across the sample plane and uniform along the out-of-plane direction (fig.9). The randomized T_c_ distribution is mathematically modelled by summing up a set of spatially varying waves with random amplitudes and phase angles. Specifically, we used the following equations:

𝑓 (2)

𝑎 (3)


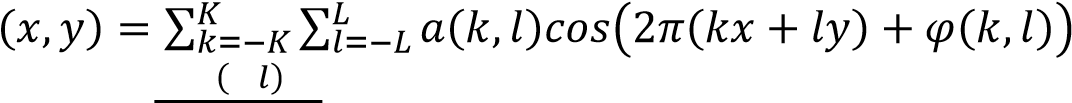

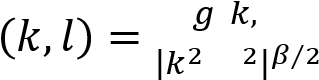
+𝑙

The function *g(k,l) h*ave a random Gaussian distribution, so that a(*k*,*l*) is a random frequencydependent amplitude function with values that taper off for higher frequencies in accordance with the spectral exponent β. The higher the value of β, the smoother the generated data will be, so that the value of β *allows to simulate* increasing spatial scales of disorder, with ever present slower variations and progressively dominant faster variations. The integer numbers K and L determine the maximum frequencies in each planar direction (K=L=40 was chosen). The phase angles *φ* in eq. (2) are sampled from uniform random distribution between -π/2 and /2. For a given T_c_ obtained from eqs. (2) and (3), the resistivity was modelled as a sharp transition. We chose a set of β *values such that the spatial scale of disorder evaluated from the Fast Fourier Transform of Tc values is* approximately 10, 15, 30, 70, 400 and 800 μm, respectively, so that this disorder scale ranges from values much smaller than the sample size to values comparable to the sample size. All the results remain unchanged if all the spatial lengths (sample size, scale of disorder, size of the Hall voltage pads) are scaled by the same factor.
